# The Overexpression of RTN4 Significantly Associated With an Unfavourable Prognosis in Patients With Lower‐Grade Gliomas

**DOI:** 10.1111/jcmm.70418

**Published:** 2025-02-19

**Authors:** Jing Feng, Lin Zhao, Huiyan Chen, Jianhai Lin, Mingchao Shang, Baoqing Xu, Xinpeng Wang, Danyu Ma, Jinping Zhou, Hu Zhao

**Affiliations:** ^1^ Department of Radiation Oncology, 900TH Hospital of Joint Logistics Support Force Fuzong Clinical Medical College of Fujian Medical University Fuzhou China; ^2^ Department of Radiation Oncology, School of Medicine, Dongfang Hospital of Xiamen University 900TH Hospital of Joint Logistics Support Force, Xiamen University Fuzhou China; ^3^ Department of Radiation Oncology, 900TH Hospital of Joint Logistics Support Force Fujian University of Traditional Chinese Medicine Fuzhou China; ^4^ Department of Neurosurgery, 900TH Hospital of Joint Logistics Support Force Fuzong Clinical Medical College of Fujian Medical University Fuzhou China; ^5^ Department of General Surgery, 900TH Hospital of Joint Logistics Support Force Fuzong Clinical Medical College of Fujian Medical University Fuzhou China; ^6^ Department of Pathology, 900TH Hospital of Joint Logistics Support Force Fuzong Clinical Medical College of Fujian Medical University Fuzhou China; ^7^ Department of Clinical Quality Management 900TH Hospital of Joint Logistics Support Force Fuzhou China; ^8^ Department of General Surgery, Dongfang Hospital of Xiamen University, School of Medicine 900TH Hospital of Joint Logistics Support Force, Xiamen University Fuzhou China; ^9^ Department of General Surgery, 900TH Hospital of Joint Logistics Support Force Fujian University of Traditional Chinese Medicine Fuzhou China

**Keywords:** bioinformatics, biomarker, gliomas, lower‐grade gliomas, prognosis, RTN4

## Abstract

Gliomas, the most prevalent primary malignancy of the central nervous system, is characterised by its high mortality rates and unfavourable prognosis. Despite extensive research, the underlying mechanisms of glioma pathogenesis remain elusive. The Cancer Genome Atlas (TCGA) and Chinese Glioma Genome Atlas (CGGA) databases provided the lower‐grade gliomas (LGG) transcriptome and related clinical data, which were downloaded separately. It was determined what the clinical data differences were between the two groups based on the median reticulon‐4 (RTN4) expression group. The R language's survminer tool was utilised to examine the variations in survival between the RTN4 high and low‐expression groups. The GeneMANIA database was searched for genes that might interact with RTN4, and these genes were then used to create extensive coexpression networks. Cox regression analysis, both univariate and multivariate, was used to filter out the independent prognostic factors influencing tumour growth. Based on independent prognostic parameters, a nomogram was created to predict prognosis. The model was assessed both internally and externally using receiver operating characteristic curve (ROC) and correcting curves. The R cibersort package was utilised to assess the level of immune infiltration abundance. We further validated our findings with clinical tissues using immunohistochemistry approaches. Statistical significance was determined using the Wilcoxon signed‐rank test, with a *p* value of < 0.05 considered significant. RTN4 expression in the tumour group was higher than in the normal group (*p* < 0.001), and a high‐expression level was linked to a poor prognosis (*p* = 0.028). Patients with elevated RTN4 expression exhibited significant differences from normal brain tissue samples when stratified analysis of LGG patients by sex or radiation treatment was performed (*p* < 0.001). The immune cell infiltration data demonstrated that the two groups' expressions of various immune cells differed, with pDC cells showing the greatest correlation (−0.421). Univariate and multivariate Cox regression study showed that RTN4, isocitrate dehydrogenase (IDH) mutation, 1p19q codeletion and nia age could be employed as independent prognostic factors for LGG, and the correction curve of the model fit well. Ultimately, clinical samples' immunohistochemistry revealed that RTN4 was markedly overexpressed in low‐grade gliomas. High RTN4 expression was strongly associated with a poor prognosis in LGG patients. RTN4 may serve as a prognostic biomarker for patients with LGG and represents a potential therapeutic target for immunotherapy in this patient population.

## 1 Introduction

Gliomas, originating from brain glial cells, is recognised as the most prevalent primary intracranial tumour, exhibiting a highly malignant nature. Its five‐year mortality rate was surpassed only by pancreatic and lung cancers, making it a significant threat to human health [1]. The diagnosis of gliomas involved the acquisition of tumour samples through resection or biopsy surgery, allowing for a comprehensive diagnosis encompassing histopathology and molecular pathology. This diagnostic process resulted in the determination of the pathological grade and molecular subtype, both crucial factors in predicting the prognosis and clinical outcomes of gliomas patients undergoing individualised treatment [2, 3]. In the 2021 classification by the World Health Organization (WHO) of central nervous system tumours, gliomas were categorised into grades 1–4, with grades 2–3 designated as lower‐grade gliomas based on their biological behaviour and prognosis [1, 4]. Although the prognosis for patients with lower‐grade gliomas was generally more favourable than that for patients with glioblastoma, an effective curative treatment remained elusive. Despite the combined application of surgery, radiotherapy and chemotherapy, the risk of recurrence persists, rendering the disease potentially life‐threatening [5, 6]. Moreover, significant heterogeneity has been observed in the survival rates of patients with LGG, ranging from a median survival of 1.7 to 13.3 years [7, 8]. Characterised by biological complexity, high recurrence rates and a poor prognosis, LGG presented significant challenges in diagnosis and prognosis. Therefore, the identification of novel diagnostic and prognostic biomarkers remains a top priority in the field.

Tumour development has been intimately associated with a vast array of biological processes, among which alterations within the endoplasmic reticulum (ER) have emerged as a novel and pivotal approach in elucidating tumour aetiology. The ER, a versatile organelle pivotal to cellular homeostasis, was ubiquitous in eukaryotic cells across virtually all mammalian species, excluding terminally differentiated erythrocytes [9, 10]. Comprising an interconnected network of diverse morphologies, the ER pervaded the cytoplasm and establishes extensive contacts with other organelles. Dysregulation of ER morphology has been intricately linked to neurological diseases and malignancies [11, 12]. Elucidating morphological changes in the ER under varying conditions, as well as the roles of regulatory factors in physiology and pathology, holds significant importance and represents crucial avenues for exploring the intricacies of tubulin encoding. Moreover, the phenotypic structure of the ER serves as a pivotal parameter for gene and drug screening [13]. The ER, being the largest organelle within a cell, encompasses the tubular and the sheath endoplasmic reticulum. The tubules themselves were constructed from two evolutionarily conserved protein families: reticulin (RTNs) and receptor expression‐enhancing proteins(REEPs) [14, 15, 16, 17]. Notably, tubuloendoplasmic reticulum expansion has been identified as a specific marker of DNA damage [18].

Reticulins constitute a class of membrane proteins that were ubiquitously present in eukaryotic organisms and primarily localise to the endoplasmic reticulum. Within the mammalian genome, four distinct genes encoding RTN1‐4 have been identified. The highly variable amino terminus of RTNs conferred species‐specific and cell‐specific functionalities, including the modulation of amyloid precursor protein processing enzymes [19, 20]. The RTN1 signature exhibited a remarkable degree of discrimination, accuracy and clinical utility in identifying the pathological complete response of triple‐negative breast cancer following neoadjuvant chemotherapy. RTN3 has been observed to be abundantly expressed in normal hepatocytes, whereas its expression is downregulated in hepatocellular carinoma (HCC) [21]. In mice, the neuroprotective effects induced by cooling are abrogated upon knockdown of RTN3 expression [22]. During neurodevelopment, RTN4 served as an inhibitory cue for endothelial cell migration within the central nervous system, thereby constraining vascular density [23]. Recently, numerous studies conducted domestically and internationally have implicated RTN4 in the pathogenesis of gastric cancer [24], colorectal cancer [25], HCC [26] and prostate cancer [27]. Prior investigations have demonstrated robust expression of RTN4 in oligodendrogliomas [28, 29]. However, the role of the RTN family in lower‐grade gliomas and its association with prognosis remain unexplored.

Therefore, we conducted a meticulous screening of differential genes from The TCGA glioma samples, encompassing both LGG and glioblastoma multiforme (GBM), subsequently intersecting these with reticulin family genes. The hub genes were subsequently determined through a rigorous process of expression profiling and survival analysis. Leveraging bioinformatics and cytofunctional methodologies, we embarked on a comprehensive investigation of the pivotal role played by these hub genes in lower‐grade gliomas, delving into a preliminary exploration of the underlying mechanisms at play.

## 2 Materials and Methods

### 2.1 Data Source and Acquisition

Nine reticulin‐related genes were retrieved from Treefam [30] (http://www.treefam.org/). Subsequently, gene expression data and clinical information were acquired from the TCGA database [31] (http://cancergenome.nih.gov/). These data were then meticulously processed using the voom function within the limma package in R software. Additionally, the CGGA database [32] (http://www.cgga.org.cn/) was downloaded for further analysis. A comprehensive dataset comprising 398 cases of LGG patients aged over 18 years was utilised for survival and prognosis verification. This analysis was facilitated by the SUV and limma packages in R software, which were employed for batch correction and data processing [32].

### 2.2 Patient Information

Postoperative tissue samples were obtained from 100 gliomas patients, including adjacent normal brain tissue specimens from 3 patients diagnosed with LGG, at the 900th Hospital of the Joint Logistics Support Force, spanning the period from January 2016 to December 2022. These samples were procured for the purpose of verifying prognostic markers. Strict inclusion criteria were applied, ensuring that only newly diagnosed gliomas patients were included in the study. For the purposes of data analysis, a subset of 80 patients with comprehensive clinical data and survival information was utilised. Diagnosis was established in accordance with the diagnostic criteria outlined by the WHO in 2016 and 2021. The mutational status of IDH was determined through the Sanger sequencing method, whereas the deletion and heterozygosity status of 1p/19q were ascertained via fluorescence in situ hybridisation technology.

Gene Expression Profiling Interactive Analysis [33] (http://gepia.cancer‐pku.cn/index.html) is a publicly available interactive web server for analysing gene expression data from cancer and normal tissues from TCGA and genotype‐tissue expression [34] (GTEx, https://commonfund.nih.gov/gtex) projects. While TCGA only contained five normal brain tissue samples, in this study, we chose the GETx database as the control. The required GTEx data, including fragments per kilobase of exon model per million mapped reads (FPKM) files, phenotypic files and clinical data for LGG tumour samples, were downloaded by visiting the official websites of GTEx and TCGA. The GTEx data underwent ID conversion using the human.gtf file as a reference, with the conversion process being executed through scripting. Additionally, the expression information for the corresponding tissue samples in GTEx was extracted, and sample numbers were prepared. Similar processing steps were carried out on the TCGA data, encompassing ID conversion and the separation of normal and tumour samples. To ensure consistency, the formats of the GTEx and TCGA data were unified, encompassing factors such as gene ID and expression amount. R or other suitable data processing tools were utilised to merge the GTEx and TCGA data. The resultant merged dataset encompassed gene expression information derived from LGG tumour samples as well as normal tissue samples sourced from both GTEx and TCGA. The integrated RESM FPKM data and phenotype data (Cohort: TCGA Target GTEX) on UCSC XENA were downloaded to make a heatmap.

### 2.3 RTN4 Expression and Survival Analysis

The expression of RTN4 was assayed using the Wilcoxon symbol‐rank test. Subsequently, the Wilcoxon sign rank test was employed to investigate the association between clinicopathological features and RTN4 expression. The median expression of RTN4 in all samples was first calculated, and then it was used as a boundary to be divided the expression levels of RTN4 in LGG patients into two categories: samples with expression levels above the median were defined as having high RTN4 expression, while those with expression levels below the median were defined as having low RTN4 expression. Other statistical methods, such as the *t*‐test, were also used to further verify whether the RTN4 expression levels in patients with LGG were significantly higher than those in the control group. Kaplan–Meier curves were generated utilising the survminer package in R software (version 4.0.2) to evaluate prognostic disparities between high and low‐expression groups of RTN4 in lower‐grade glioma. The prognostic parameter focused on was Overall Survival (OS), and Cox regression analysis was conducted to assess statistical significance. A *p* value < 0.05 denoted a statistically significant difference. Hazard Ratio (HR) values greater than 1 were interpreted as risk factors, while HR values less than 1 were considered protective factors. The entire dataset was processed using R software and Adobe Photoshop CC for graphical representation.

### 2.4 Analysis of Immune Infiltration

The RNAseq data in the TCGA database, specifically the level 3 HTSeq‐FPKM format data pertaining to LGG samples, was utilised. These data, originally in FPKM format, were subsequently converted into transcripts per kilobase of exon model per million mapped reads (TPM) format and underwent log2 transformation. Duplicate samples were meticulously removed, and the molecular signatures of tumour‐immune interactions were assessed utilising the GSVA package in R software. Furthermore, a rigorous analysis was conducted to investigate the correlation between RTN4 expression levels and the abundance of 24 tumour infiltrative immune subsets.

### 2.5 Gene Set Enrichment Analysis (GSEA)

GSEA was employed to elucidate the biological pathway underlying differentially expressed genes between high and low RTN4 expression groups [35]. Subsequently, RNAseq data (level 3) and the associated clinical information of LGG were thoroughly analysed using the GSVA package in R software. The parameter method = ‘ssgsea’ was specifically chosen for this analysis. Finally, the correlation between genes and pathway scores was meticulously examined using Spearman correlation. A *p* value < 0.05 was deemed statistically significant.

### 2.6 Protein–Protein Interaction Networks (PPI) Construction

Genes potentially interacting with RTN4 were retrieved from the GeneMANIA database [36] (https://genemania.org/) and subsequently utilised to identify a comprehensive coexpression network.

### 2.7 Nomogram Diagram Construction

Based on the outcomes of the univariate Cox regression model, a nomogram prediction model was constructed to accurately forecast the 1‐, 3‐ and 5‐year overall survival rates of LGG patients. The proportional risk hypothesis was rigorously tested and Cox regression analysis was conducted utilising the survival package in R software. Furthermore, the rms package was employed for the comprehensive analysis and visualisation of the nomogram. Differences were deemed statistically significant if *p* < 0.05.

### 2.8 Immunohistochemistry Analysis

A comprehensive collection of surgical specimens was undertaken, encompassing a total of 80 patients with LGG and 3 cases of adjacent normal brain tissue from grade 1 glioma, sourced from the 900th Hospital of the Joint Logistics Support Force. These specimens were meticulously fixed using a 40 g/L formaldehyde solution and embedded in conventional paraffin, following which they were precisely sliced into 4 μm thick sections and stained with HE for histological analysis. Immunohistochemical staining of RTN4 was subsequently performed utilising the EliVision method, with the results being carefully observed under a light microscope. The staining process employed an anti‐RTN4 polyclonal antibody (1:400 dilution), sourced from Abcam, UK (Catalogue Number: ab180847), in conjunction with a non‐biotin universal two‐step immunohistochemistry kit (mouse/rabbit enhanced polymer detection system, PV‐9000), acquired from Beijing Zhongshan Jinqiao Biotechnology Co. Ltd.

For the interpretation of staining results, RTN4 positivity was identified as the presence of brownish‐yellow granules within the cytoplasm. Cells exhibiting dark brown cytoplasm were classified as strong intensity, while those with yellow or brown cytoplasm were designated as medium intensity. Cells with a pale‐yellow cytoplasm or faint staining were categorised as weak intensity. Cells lacking cytoplasmic staining were considered negative. The expression level of RTN4 was quantitatively assessed using the histochemical score (H‐score) method, calculated as follows: H‐score = (weak intensity percentage of cells × 1) + (medium intensity percentage of cells × 2) + (strong intensity percentage of cells × 3).

### 2.9 Statistical Methods

The OS rates of the immunohistochemically high‐expression and low‐expression groups were comparatively analysed using the Kaplan–Meier method. Significant differences between the groups were subsequently detected via the Wilcoxon signed‐rank test. Potential risk factors were identified through univariate Cox regression analysis. Statistical significance was ascribed to findings with a *p* value less than 0.05. All data were processed utilising the statistical software R (version 4.0.2).

## 3 Results

### 3.1 RTN4 Was Significantly Overexpressed in LGG and Suggested a Poor Prognosis in Glioma Patients

**FIGURE 1:**
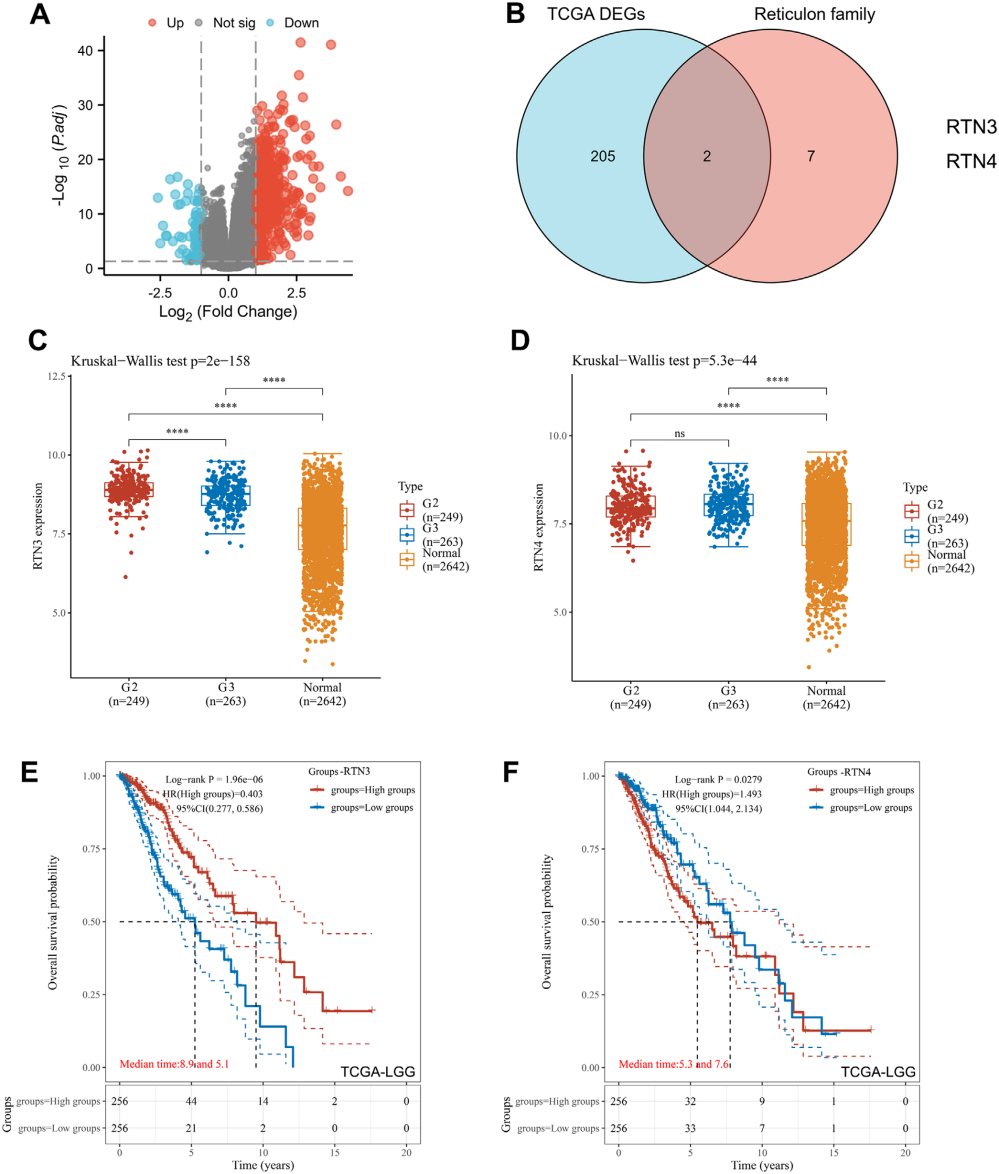
Gene selection. Expression level and prognostic analysis of TCGA‐LGG (A). Venn diagram of differentially expressed genes (DEGs) and reticulin genes (B). Expression level of RTN3 in G2, G3 and normal tissues (ns *p* > 0.05, ****p < 0.0001) (C). (D) Expression levels of RTN4 in G2, G3 and normal tissues. (E) Overall survival of RTN3 in LGG Kaplan–Meier analysis. (F) Overall survival of RTN4 in LGG Kaplan–Meier analysis.

Based on the criteria of an adjusted *p* value less than 0.05 and a log2 fold change greater than 1.5, the transcriptional profiling of TCGA‐LGG identified 207 differentially expressed genes (DEGs) (Figure 1A). Among these, the intersection with the reticulin gene family revealed two genes: RTN3 and RTN4 (Figure 1B). Initially, we validated the expression patterns of these two genes in LGG by contrasting the transcriptome data of LGG tissues in the TCGA database with those of normal brain tissues in the GTEx database. The findings demonstrated a significant upregulation of RTN3 and RTN4 expression in LGG (Figure 1C,D). Subsequently, we evaluated the prognostic significance of RTN3 and RTN4 in LGG. Survival analysis (Figure 1E,F) revealed that high RTN4 expression (*p* = 0.028) correlated with poorer overall survival in LGG patients, whereas high RTN3 expression (*p* < 0.001) was associated with improved overall survival. These results indicate that RTN4, rather than RTN3, could potentially serve as a therapeutic target and adverse prognostic biomarker in LGG patients. Notably, the expression levels of RTN3 and RTN4 did not exhibit a significant association with the survival prognosis of patients with GBM (*p* > 0.05) (Figure S1).

### 3.2 Clinical Relevance

**FIGURE 2:**
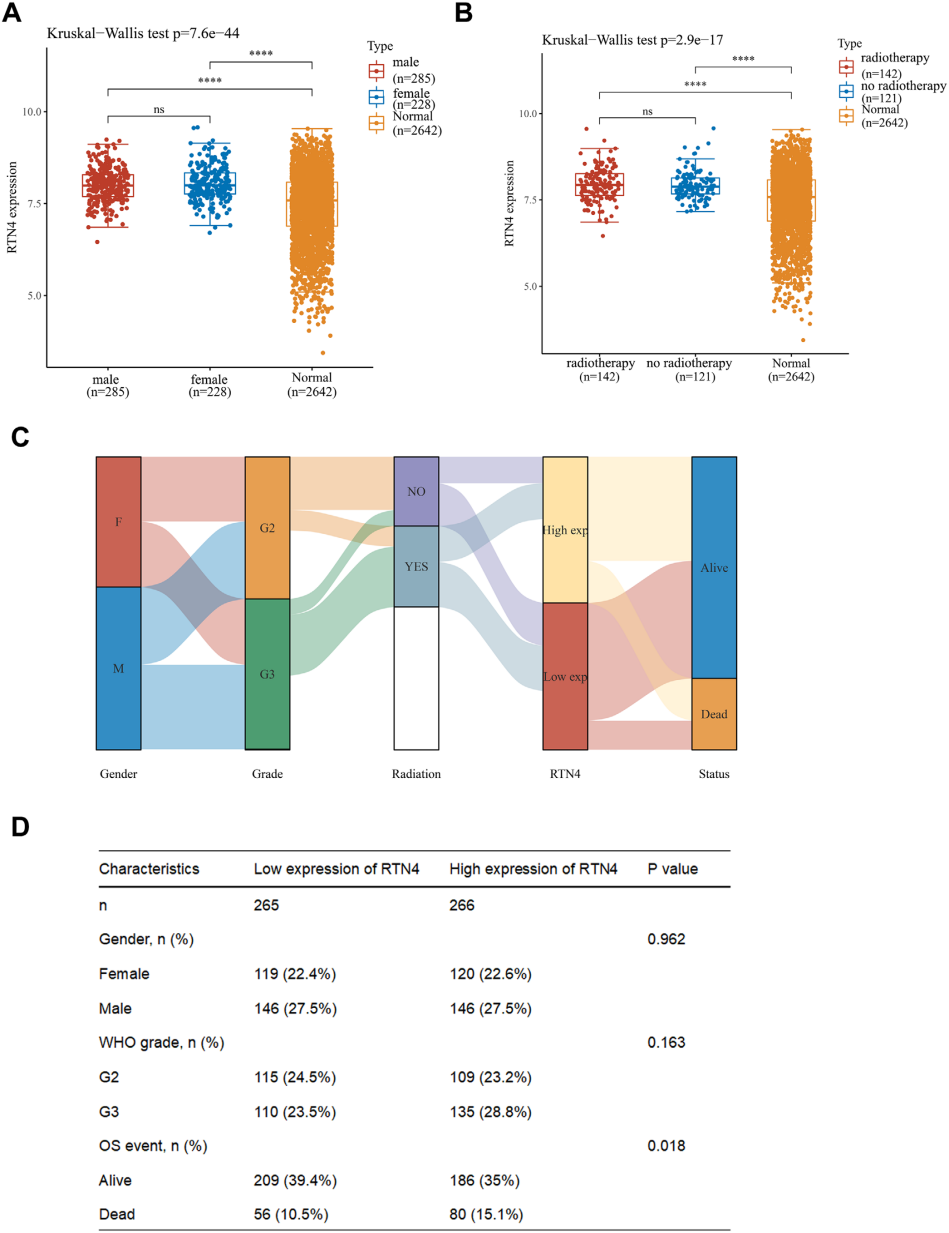
Expression of RTN4 in LGG. (A) Expression levels of RTN4 in male, female and normal tissues (ns *p* > 0.05, ****p < 0.0001). (B) Expression levels of RTN4 in radiotherapy, non‐radiotherapy and normal tissues (ns *p* > 0.05, ****p < 0.0001). (C) Sankey diagram. (D)The chi‐square test with RTN4 expression in LGG.

Stratified analysis of LGG patients further revealed no significant differences in the frequency of high RTN4 expression among patients stratified by sex or radiotherapy receipt, but significant differences were observed in comparison to normal brain tissue samples (Figure 2A,B). Additionally, a Sankey plot demonstrated robust associations between sex, tumour grade, survival status and RTN4 expression (Figure 2C). We performed the chi‐square test and found that only survival status was significantly associated with RTN4 expression in LGG (Figure 2D). Baseline data indicated significant correlations between high and low RTN4 expression with IDH mutation status, 1p19q codeletion, pathological type and age (Table S1). Univariate Cox regression analysis was employed to assess the prognostic significance of RTN4 expression in LGG patients. This analysis revealed that high RTN4 expression was significantly associated with shorter overall survival time (HR 1.502, 95% CI = 1.052–2.145, *p* = 0.025). Furthermore, other variables predictive of poor survival included age at diagnosis, IDH mutation status, 1p19q codeletion and pathological type (*p* < 0.05) (Table S2).

### 3.3 Survival Analysis

**FIGURE 3:**
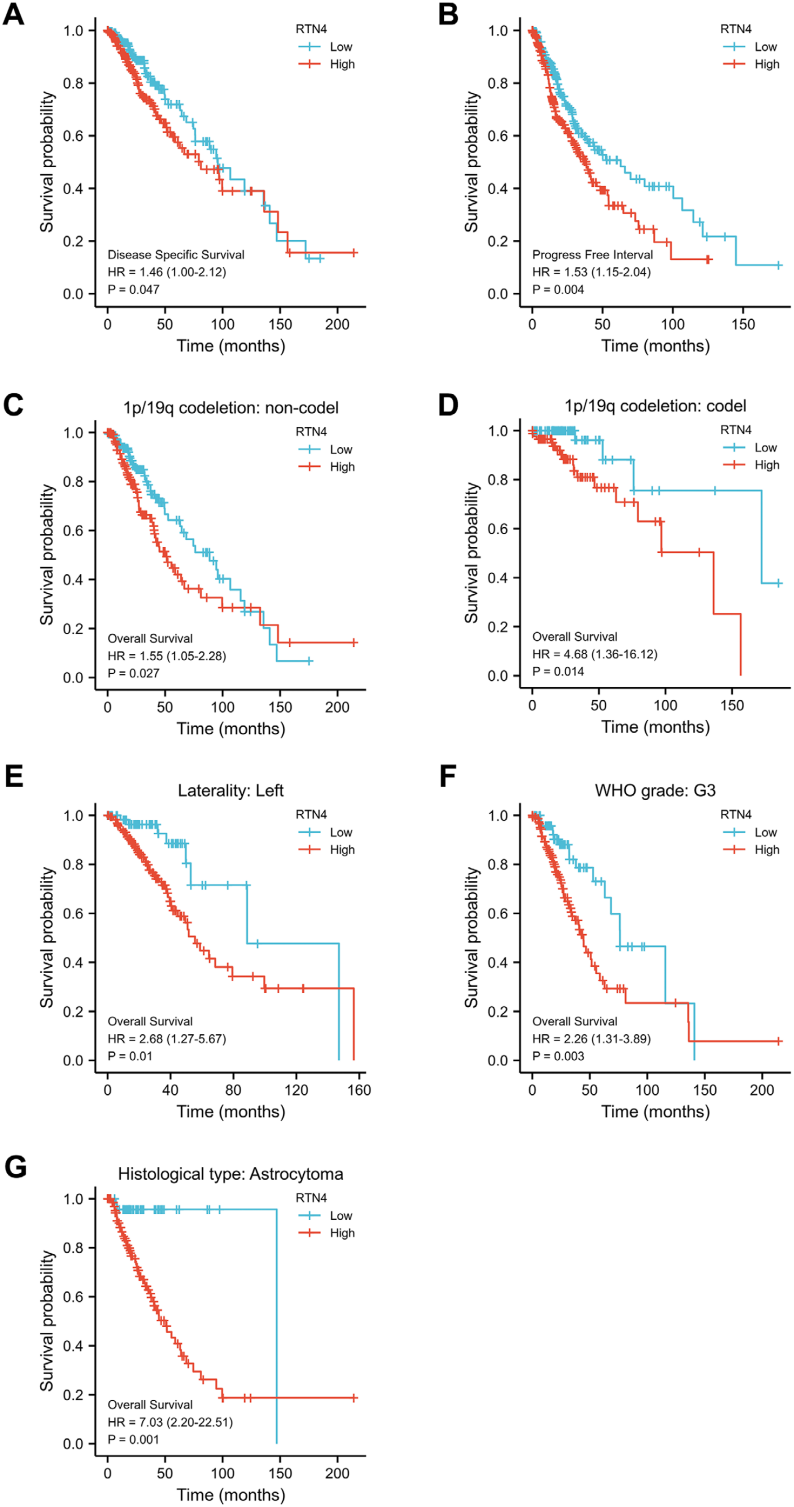
Survival analysis of RTN4 in LGG. (A) DSSKaplan–Meier analysis of RTN4 in LGG. (B) PFSKaplan–Meier analysis of RTN4 in LGG. (C) Kaplan–Meier analysis of overall survival of RTN4 in 1p19q uncombined deletion LGG. (D) Overall survival of RTN4 in 1p19q combined deletion LGG with Kaplan–Meier analysis. (E) Overall survival of RTN4 in LGGs with lesions located to the left of the midline Kaplan–Meier analysis. (F) Overall survival of RTN4 in WHO3‐grade LGG Kaplan–Meier analysis. (G) Kaplan–Meier analysis of overall survival of RTN4 in LGG with pathological type Astrocytoma.

To delve deeper into the relationship between RTN4 expression and patient survival status, and to identify potential prognostic markers, survival analysis was conducted utilising the TCGA database. Among the 532 LGG sequencing datasets retrieved from the TCGA, 531 contained prognostic information. Genes exhibiting expression levels exceeding the median in these cancerous tissues were deemed to be highly expressed, and conversely, those below the median were considered to have low expression. Kaplan–Meier survival curves were plotted for the RTN4 high‐ and low‐expression groups, based on the overall survival data from the TCGA‐LGG database, and a log‐rank test was subsequently performed. The findings revealed a significant association between RTN4 gene expression and Disease‐Free Survival (DFS) as well as Progression Free Interval (PFI) in LGG patients (*p* = 0.047, *p* = 0.004). Specifically, patients with or without 1p19q codeletion exhibited a notable correlation between high RTN4 expression and OS (*p* = 0.027, *p* = 0.014). Additionally, in surviving patients, a significant linkage was observed between high RTN4 expression and OS (*p* = 0.01). Furthermore, in patients with WHO grade III astrocytoma, both the pathological type and high RTN4 expression were significantly associated with OS (*p* = 0.003, *p* = 0.001) (Figure 3A–G). Consistently, RTN4 gene expression was significantly associated with OS in LGG patients from the CGGA database as well (*p* = 0.048) (Figure S2A).

### 3.4 RTN4 Is Correlated With Immune Infiltration in LGG

Figure S3A demonstrates a positive correlation between RTN4 expression and the infiltration of mast cells, TFH cells, Th1cells, T cells, B cells, NK CD56bright cells, TTh2 cells, Tcm cells, NK CD56dim cells and Tem cells, while exhibiting a negative correlation with the infiltration of pDCs, CD8+ T cells, DCs and Tgd. Statistically significant differences in enrichment scores were observed between the high‐ and low‐RTN4 expression groups for pDCs, CD8+ T cells, TFH cells and mast cells (*p* < 0.001) (Figure S3B). Specifically, the correlation analysis revealed significant associations between RTN4 expression and the infiltration of TFH cells (*r* = 0.276, *p* < 0.001), mast cells (*r* = 0.271, *p* < 0.001), pDCs (*r* = 0.421, *p* < 0.001) and CD8+ T cells (*r* = 0.282, *p* < 0.001) (Figure S3C–F). These findings suggest that RTN4 may serve as a potential immunomodulator in LGG. Notably, a robust correlation was observed between pDCs and RTN4 expression, whereas the numbers of CD8+ T cells, TFH cells and mast cells exhibited a more modest correlation with RTN4 expression.

### 3.5 Enrichment Analysis of RTN4‐Related Genes in LGG

**FIGURE 4:**
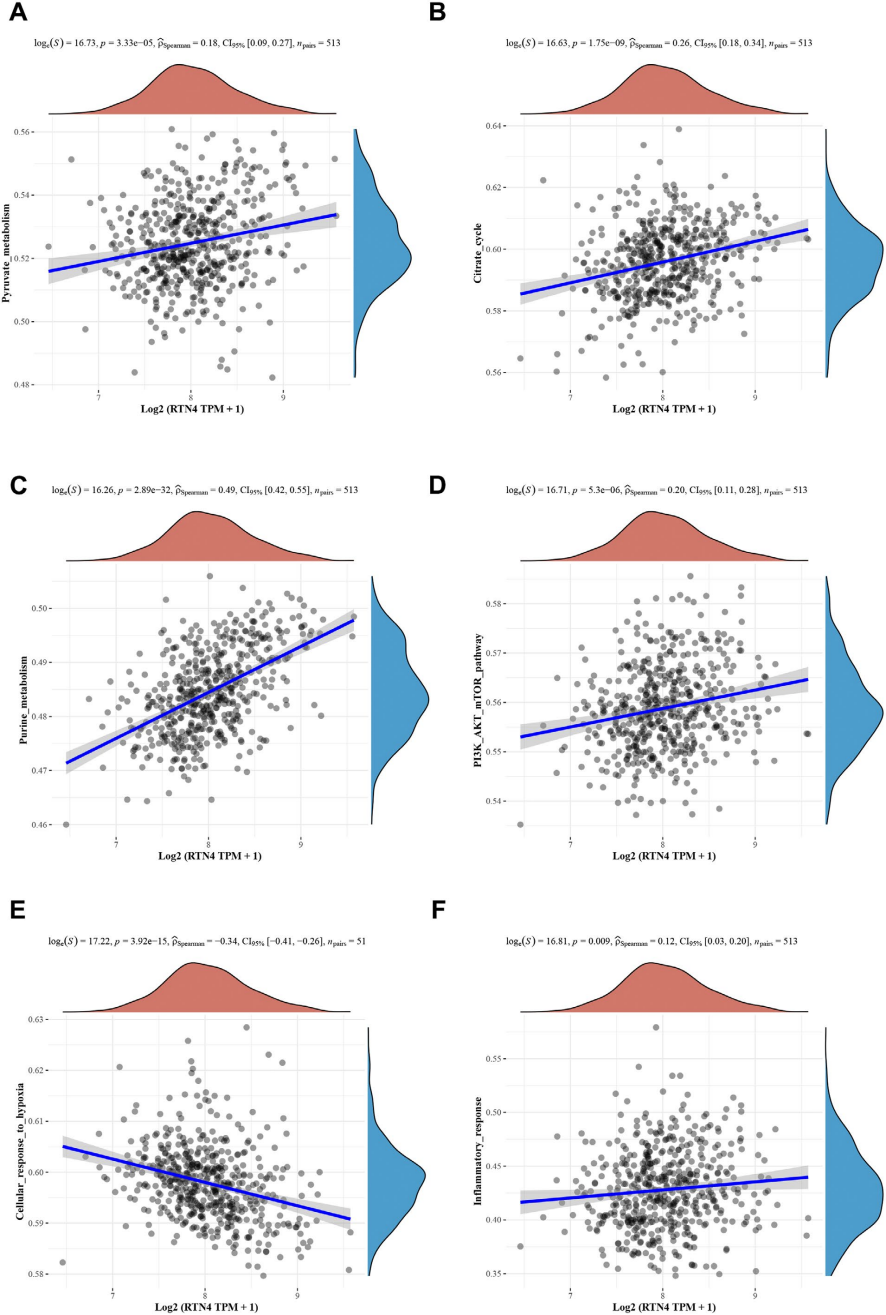
Functional enrichment analysis of related genes of RTN4 in LGG.

We further delved into the mechanistic underpinnings of RTN4's role in LGG from a bioinformatics standpoint. By compiling gene sets inherent to pertinent pathways [37], the enrichment score of each sample within each respective pathway was systematically computed utilising the ssGSEA algorithm, thereby elucidating the intricate linkage between samples and their corresponding pathways. An appropriate multiple hypothesis correction method, such as false discovery rate (FDR) control, was utilised to adjust the *p* values and account for the multiple testing involved in our pathway enrichment analysis. Furthermore, gene‐pathway relationships were elucidated by assessing the correlation between gene expression and pathway scores. Notably, our enrichment analysis revealed that six distinct pathways (*p* < 0.05, NES > 2), specifically pyruvate metabolism, the citrate cycle, purine metabolism, the PI3K_AKT_mTOR pathway, the cellular response to hypoxia and the inflammatory response, exhibited a significant association with elevated RTN4 expression (Figure 4A–F).

### 3.6 RTN4 Expression in the PPI Network in LGG

To gain insights into the potential biological functions mediated by RTN4, we conducted an exhaustive screening of RTN4R and LINGO1 in the GeneMANIA database. Our focus encompassed a wide range of interaction types, including physical interactions, coexpression patterns, predictions, colocalisation, pathway involvement, genetic interactions and shared protein domains. This meticulous analysis led to the identification of a comprehensive network comprising 20 proteins that interact with RTN4. These proteins are RTN3, NUS1, RTN1, RTN2, NDN, ARHGDIA, TMEM170A, NGFR, HSPA4L, LRCH4, RTN4IP1, ARL1, ATL3, IGFBP7, CALM1, WASHC4, BCL2L1 and UQCRC1.

**FIGURE 5:**
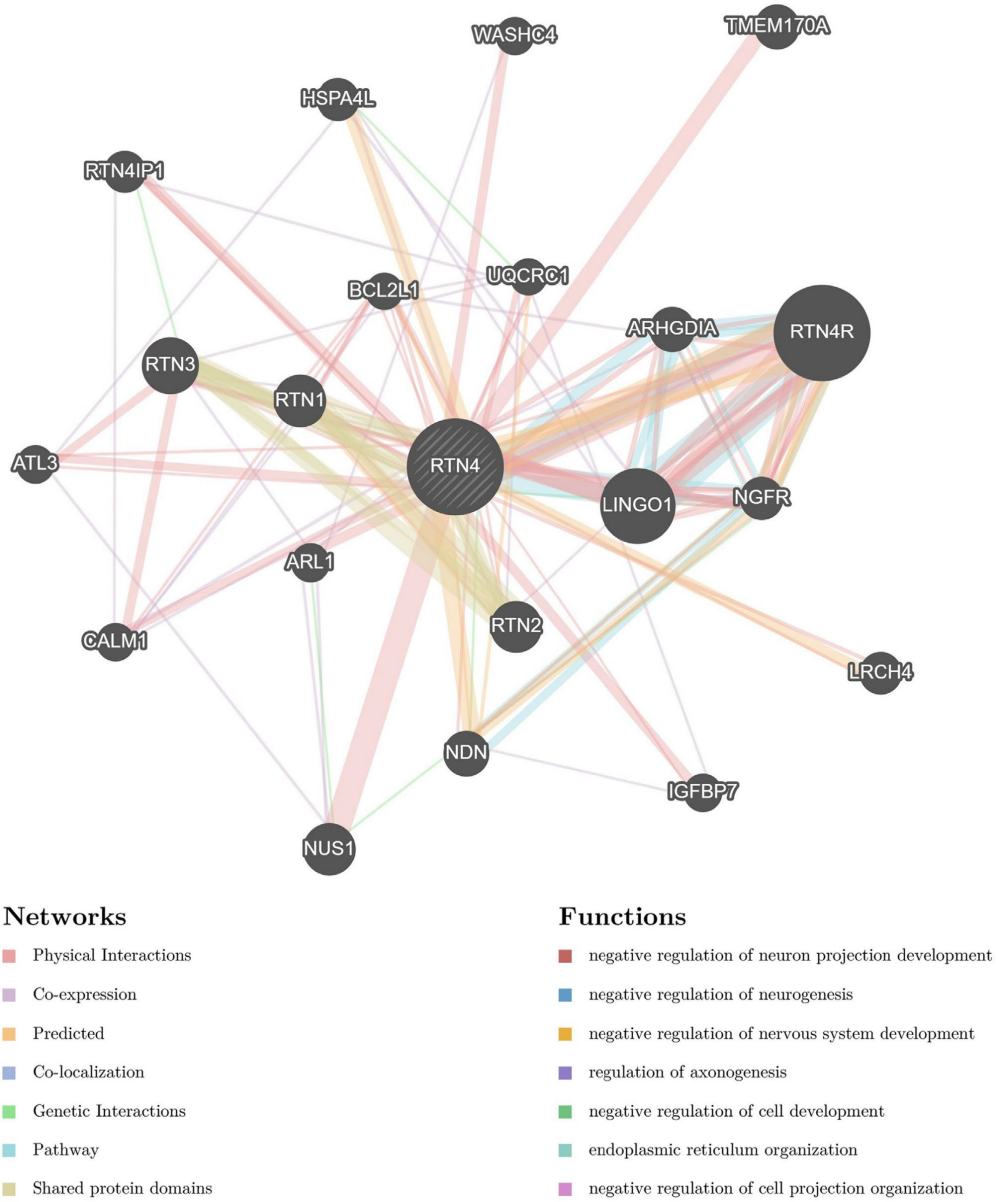
RTN4 PPI network in LGG.

Subsequently, we constructed a network diagram to visually represent these intricate interactions (Figure 5). Beyond the mere depiction of the network, we delved deeper into the biological significance of the identified proteins. Notably, several of these proteins are implicated in processes such as the negative regulation of cell development. For instance, RTN3 and RTN1, both reticulon family members, are known to play crucial roles in endoplasmic ER organisation and maintenance. Similarly, ARHGDIA, which interacts with RTN4, functions in the regulation of cytoskeletal organisation and cell migration. The presence of NGFR suggests potential roles in neuronal differentiation and survival, while IGFBP7, a member of the insulin‐like growth factor binding protein family, is involved in cell proliferation and differentiation.

Collectively, the biological functions attributed to this network encompass a diverse array of processes, including but not limited to the negative regulation of cell development, endoplasmic reticulum organisation and the negative regulation of cell projection organisation. These findings provide a deeper understanding of the potential roles of RTN4 and its interacting partners in biological processes relevant to LGG.

### 3.7 Correlations Between Clinical Characteristics of LGG Patients and RTN4 Expression

**FIGURE 6:**
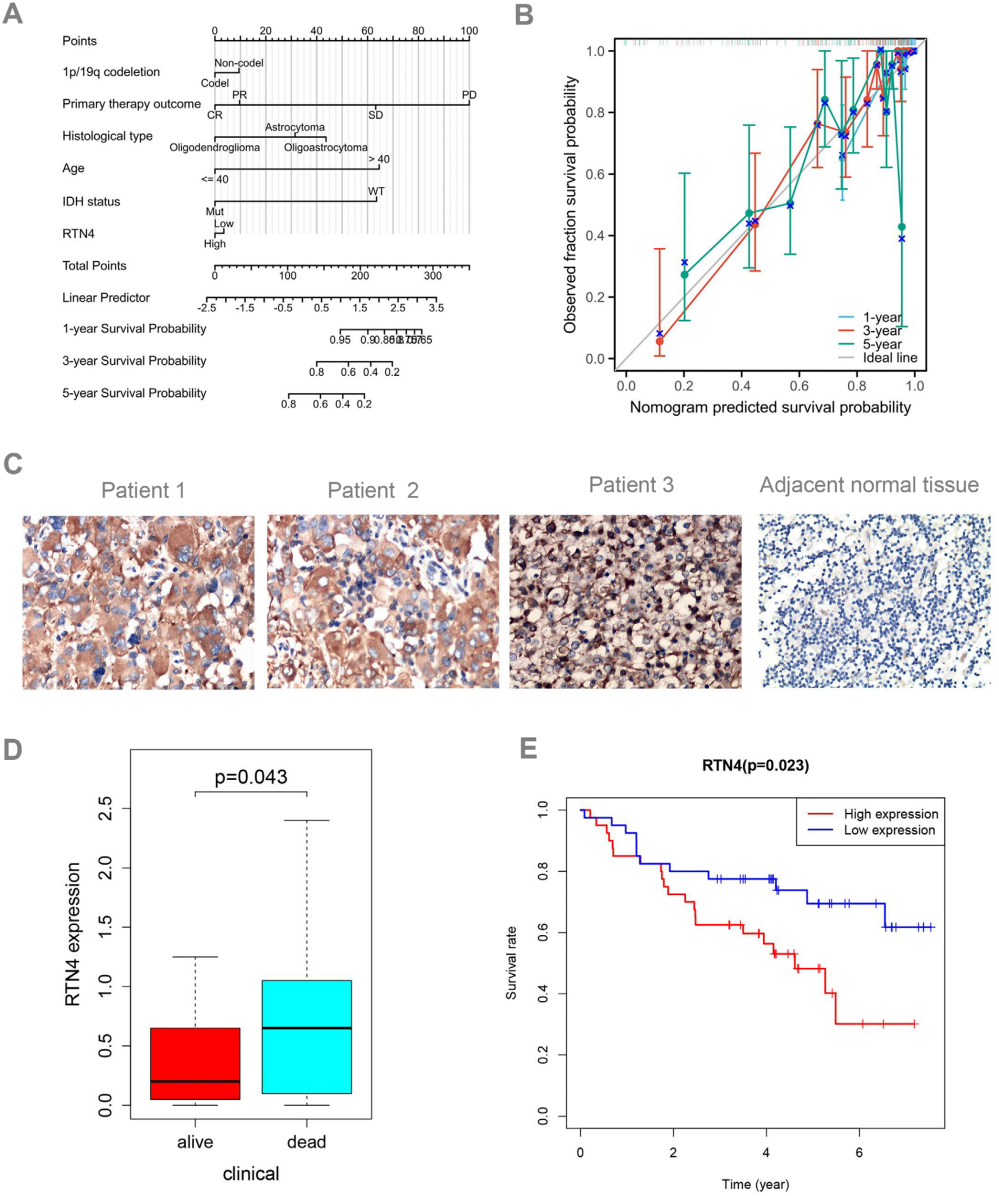
Correlation between RTN4 expression and clinical characteristics of LGG (A) (B) was significantly associated with high expression of RTN4 in LGG in TCGA, (C) (D) was significantly associated with high expression of RTN4 in LGG in clinical samples (E) and poor OS with high expression of RTN4 in clinical samples.

Among the downloaded TCGA‐LGG sequencing data, 509 samples possessed both clinical information and overall survival data. A univariate Cox regression analysis was subsequently conducted to investigate the association between RTN4 expression and OS in LGG patients, as well as its correlation with other clinical features pertinent to these patients, as detailed in Table S1. The univariate correlation analysis revealed significant associations between IDH mutation (HR = 0.155, *p* < 0.01), 1p19q codeletion (HR = 2.562, *p* < 0.01), age (HR = 2.868, *p* < 0.01) and RTN4 expression (HR = 1.502, *p* = 0.025) with OS. Utilising the findings from the univariate Cox analysis, a prognostic nomogram tailored for LGG patients was formulated to assess the independent prognostic factors that influence OS. The aggregate score derived from these factors constitutes the total score, which serves as a predictor for the patient's 1‐, 3‐ and 5‐year OS rates (Figure 6A,B). Furthermore, an analysis of the CGGA database revealed a correlation between high RTN4 expression and both IDH mutation (*p* = 0.037) and 1p19q codeletion (*p* = 0.044). However, there was no statistical significance between G2 and G3 (*p* = 0.057), and our cohort also showed no statistical significance between WHO2 and WHO3 (*p* = 0.119) (Figure S2B).

## 4 RTN4 Was Highly Expressed in Our Cohort

To further validate the expression of RTN4 in our cohort, we assayed its expression in our clinical samples and discovered that RTN4 was significantly overexpressed in lower‐grade gliomas (Figure 6C). Additionally, our findings demonstrated a correlation between elevated RTN4 expression and patient survival (*p* = 0.043) (Figure 6D). Furthermore, a higher level of RTN4 expression was associated with poorer overall survival (OS) in our cohort (Figure 6E). Univariate Cox analysis was employed to assess the prognostic significance of RTN4 expression in LGG patients. The univariate analysis further revealed a significant association between high RTN4 expression and shortened OS (HR = 2.59, 95% CI = 1.35–4.96, *p* = 0.004) (Table S3). Other variables that were associated with worse survival encompassed age and the status of complete resection (Table S3).

## 5 Discussion

Glioma poses a significant threat to human health, with recurrence and rapid progression being the principal causes of mortality in patients with LGG. The intricate interplay of numerous genes and complex regulatory networks played a pivotal role in the onset and progression of glioma, thus necessitating the urgent identification of novel biomarkers and therapeutic targets to enhance the prognosis of glioma patients. In recent years, advancements in gene sequencing and holographic technology have greatly enhanced our understanding of the potential pathogenesis underlying glioma, thereby paving the way for improved diagnostic and therapeutic approaches.

The endoplasmic reticulum's morphology and physiological roles have recently drawn more interest in cancer research. The endoplasmic reticulum, a continuous membrane system, relies on integral membrane proteins for the formation of its tubular network [14]. The absence of these proteins, which stabilised membrane curvature at the edges of tubular cross‐sections and lamellar regions, resulted in a reduction of the endoplasmic reticulum's tubular network [38]. The morphology of the endoplasmic reticulum was intricately linked to its physiological functions and various neurological disorders [39]. Initially, we screened two reticulin‐related genes, RTN3 and RTN4, from the TreeFam and TCGA differential gene databases, utilising the LGG database from the TCGA for clinical correlation analysis. The Kaplan–Meier curve further suggests that RTN4 possesses significant prognostic and clinical diagnostic and therapeutic value.

High expression of RTN4 was correlated with proliferation, tumorigenesis and patient survival in multiple types of human cancers. RTN4 exhibited a profound association with the pathogenesis of gastric cancer [24], colorectal cancer [25], HCC [26], prostate cancer [27] and numerous other malignancies. The preliminary bioinformatics analysis of RTN4 in lower‐grade gliomas has elicited several pertinent inquiries. Firstly, what was the expression pattern of RTN4 in LGG? Secondly, what were the underlying mechanisms involved in RTN4's function in LGG? Thirdly, what was the role of RTN4 within the tumour microenvironment? In pursuit of addressing these queries, we have systematically analysed the role of RTN4 in LGG, leveraging a comprehensive set of informatics tools. Therefore, we focused on RTN4.

In a study conducted by Chi et al. [24], immunohistochemical analysis was performed on histological specimens from 95 patients with primary gastric cancer to assess the correlation between RTN4 expression and clinical variables as well as prognosis. The findings revealed that 57.9% of the patients exhibited high RTN4 expression, which was significantly associated with clinical advancement and shorter survival durations. Notably, RTN4 emerged as an independent prognostic indicator for gastric cancer. Furthermore, RTN4 plays a pivotal role in the proliferative capacity of colorectal cancer cells. Xue et al. [25] employed lentivirus‐mediated RNA interference to attenuate the proliferative rate of colorectal cancer cells with silenced RTN4‐C. Flow cytometry analysis demonstrated that downregulation of RTN4 in these cancer cells led to cell cycle arrest primarily in the G0/G1 phase, particularly in the sub‐G1 phase, indicative of apoptotic cells. Our investigation revealed that in LGG, RTN4 expression is prognostic, with high‐expression levels being predictive of poor outcomes, irrespective of LGG grade. These observations suggested that RTN4 fulfilled an oncogenic‐like role in the development and progression of lower‐grade gliomas.

Many studies indicated that RTNs participate in protein trafficking and membrane structural morphogenesis or stabilisation, play a role in cell division, form channels or transportors in ER, regulate apoptosis, modulate β‐site APP cleaving enzyme 1 (BACE1) negatively, inhibit neuron regeneration and so on.

Pathak et al. reported that downregulation of RTN4 attenuated sphingomyelin synthesis and disrupted AKT localisation within the plasma membrane. Furthermore, the knockdown of RTN4 retarded the proliferation of cancer cells and mouse tumour xenografts in vitro. Additionally, this knockdown perturbed tubulin stability, thereby potentiating the cytotoxic effects of chemopaclitaxel on cancer cells, both in vitro and in vivo [40]. Furthermore, RTN4 is intricately implicated in neuronal responses to hypoxia and oxidative stress, potentially contributing to neuroblastoma formation [41].

In this article, we delved deeper into the functions and associated pathways of RTN4 through enrichment analysis. The findings suggested that RTN4 might be implicated in the PI3K_AKT_mTOR pathway, cellular responses to hypoxia and inflammatory responses in the genesis and progression of tumours. GSEA further corroborated that RTN4 is involved in LGG progression via tumour‐related signalling pathways and inflammatory responses, indicating that targeted modulation of RTN4 could serve as a potential therapeutic strategy for LGG treatment.

RTN4 is implicated in the regulation of macrophages in inflammatory diseases. Kimura et al. [42] reported that RTN4‐B is integral to the immune response elicited by nucleic acid‐sensitive TLRs. Our findings demonstrated that the expression level of RTN4 in LGG was significantly associated with pDC immune infiltration (*r* = 0.421, *p* < 0.001) (Figure S3E). Furthermore, GSEA unveiled a correlation between RTN4 expression and the inflammatory response (Figure 4F).

Subsequently, we delved into the function of RTN4 in lower‐grade glioma cells by analysing 100 LGG patients and 3 specimens of normal brain tissue from our institution. Immunohistochemistry was utilised to detect the expression of RTN4 in 80 LGG patients and 3 specimens of normal brain tissue, validating the outcomes of the bioinformatics analysis. Notably, RTN4 exhibited relatively high‐expression in LGG patients, and those with elevated RTN4 expression displayed a poorer prognosis. But there was no significant RTN4 expression in different stages of gliomas. These observations suggest that RTN4 may function as a proto‐oncogene, facilitating the transition of brain tissue from a normal state to a cancerous one and is intricately linked to clinical prognosis. These findings align with the results of bioinformatics studies, further corroborating the role of RTN4 as a protumour gene in the genesis and progression of glioma.

In terms of clinical settings, to use RTN4 as a biomarker for prognosis in patients with LGG, quantification and criteria of RTN4 overexpression level were needed. Firstly, to establish a reliable quantification method for RTN4, advanced proteomic technologies such as immunohistochemical staining, mass spectrometry or quantitative PCR may be beneficial to adopt. Secondly, in determining the criteria for RTN4 overexpression, an absolute threshold could be based on predefined levels of RTN4 expression that were statistically significantly associated with poorer prognosis in a large cohort of LGG patients. Furthermore, validation of these quantification methods and criteria in independent patient cohorts was essential to confirm their robustness and reproducibility. Lastly, given the complexity and heterogeneity of LGG, it is likely that RTN4 levels may not be the sole determinant of prognosis. Therefore, integrating RTN4 with other relevant biomarkers and clinical parameters through multivariate analysis could provide a more comprehensive picture of disease progression and response to treatment.

However, several limitations were evident in this study. Firstly, despite observing the phenomenon where the RTN4 gene promotes glioma progression, which concurred with the findings of bioinformatics analysis, the precise biological mechanism underlying this process remains elusive. Specifically, the question of whether overexpression of the RTN4 gene actually drives LGG progression by modulating the PI3K/Akt signalling pathway and inflammatory response, ultimately altering the tumour microenvironment, remains unanswered. Additional experiments are imperative to address these queries. Secondly, the clinical samples of LGG patients utilised in this study exhibited bias, necessitating the evaluation of expression disparities among samples from different centres in a larger cohort. Nevertheless, we have, for the first time, reported the aberrant expression of RTN4 in LGG cells and its pivotal role in facilitating the growth of these cells, providing valuable insights for the potential utilisation of RTN4 as a diagnostic, therapeutic and prognostic marker in LGG.

In summary, based on data derived from two databases, namely TCGA and CGGA, along with clinical samples, we have identified RTN4 as a potential oncogene in lower‐grade gliomas.

## Author Contributions

**Jing Feng:** conceptualization (equal), funding acquisition (equal), methodology (equal), project administration (equal), validation (equal), writing – original draft (equal), writing – review and editing (equal). **Lin Zhao:** conceptualization (equal), data curation (equal), formal analysis (equal), methodology (equal), resources (equal), validation (equal), visualization (equal). **Huiyan Chen:** data curation (equal), methodology (equal), writing – original draft (equal). **Jianhai Lin:** data curation (equal), methodology (equal), software (equal), validation (equal), writing – original draft (equal). **Mingchao Shang:** data curation (equal), investigation (equal), methodology (equal), validation (equal). **Baoqing Xu:** formal analysis (equal), methodology (equal), validation (equal). **Xinpeng Wang:** data curation (equal), methodology (equal), writing – original draft (equal). **Danyu Ma:** data curation (equal), validation (equal). **Jinping Zhou:** conceptualization (equal), funding acquisition (equal), project administration (equal), resources (equal), writing – original draft (equal). **Hu Zhao:** conceptualization (equal), data curation (equal), funding acquisition (equal), methodology (equal), resources (equal), validation (equal), writing – review and editing (equal).

## Ethics Statement

The study was approved by the Ethics Committee of the 900th Hospital of the Joint Logistics Support Force (Approval Number 2023‐062).

## Consent

The patients/participants provided their written informed consent to participate in this study.

## Conflicts of Interest

The authors declare no conflicts of interest.

## Supporting information


**Figure S1.** Survival and expression analysis of RTN3 and RTN4 in GBM.

**Figure S2.** Expression of RTN4 in LGG in CGGA. (A) High RTN4 expression in CGGA is associated with poorer OS. (B) Correlation between RTN4 expression and clinical features.

**Figure S3.** Correlation analysis between RTN4 expression and immune cell infiltration (A) Correlation between RTN4 expression and different immune cell infiltration in LGG (B) RTN4 expression. (C–F) Correlation between RTN4 expression and abundance of tumour‐infiltrating immune cells in LGG.

**Table S1.** Patient characteristics in TCGA database.

**Table S2.** Univariate Cox analysis evaluating independently predictive ability of RTN4 for OS in TCGA database.

**Table S3.** Univariate Cox analysis evaluating independently predictive ability of RTN4 for OS in clinical samples.

## Data Availability

The data that support the findings of this study are available from the corresponding author upon reasonable request.
