# Supplementary figures and images for: The Overexpression of RTN4 Significantly Associated With an Unfavourable Prognosis in Patients With Lower‐Grade Gliomas
Source: J Cell Mol Med. 2025 Feb 19;29(4):e70418. doi: 10.1111/jcmm.70418 (PMC11837034; doi:10.1111/jcmm.70418)

**A**

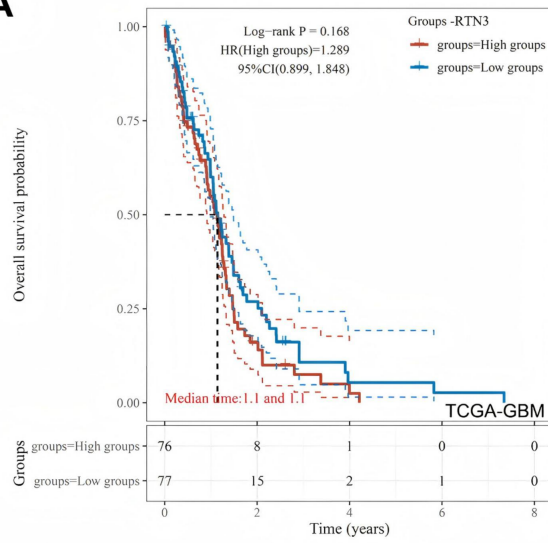

**B**

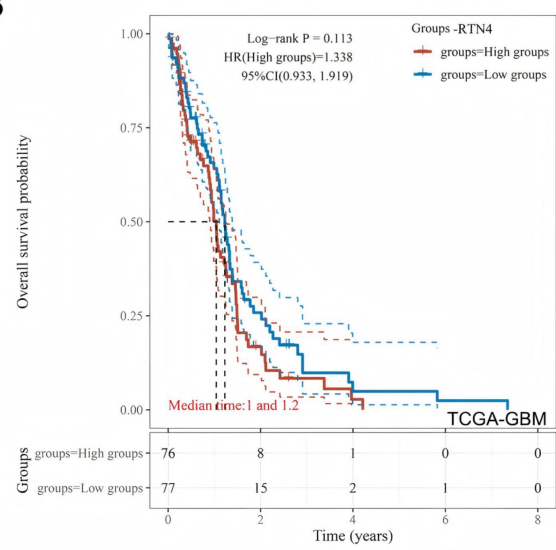

Supplement: Supplementary file 1 — Figure S1. Survival and expression analysis of RTN3 and RTN4 in GBM. [file JCMM-29-e70418-s006.pdf]

**A**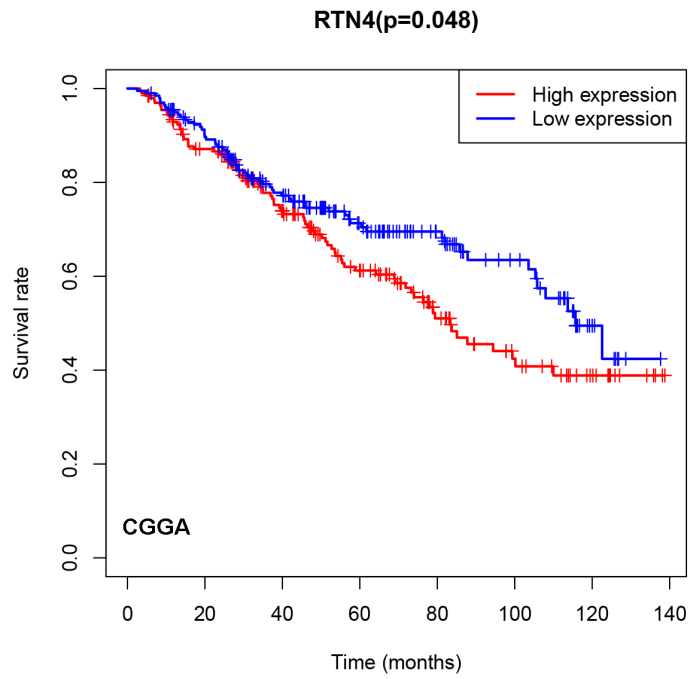**B**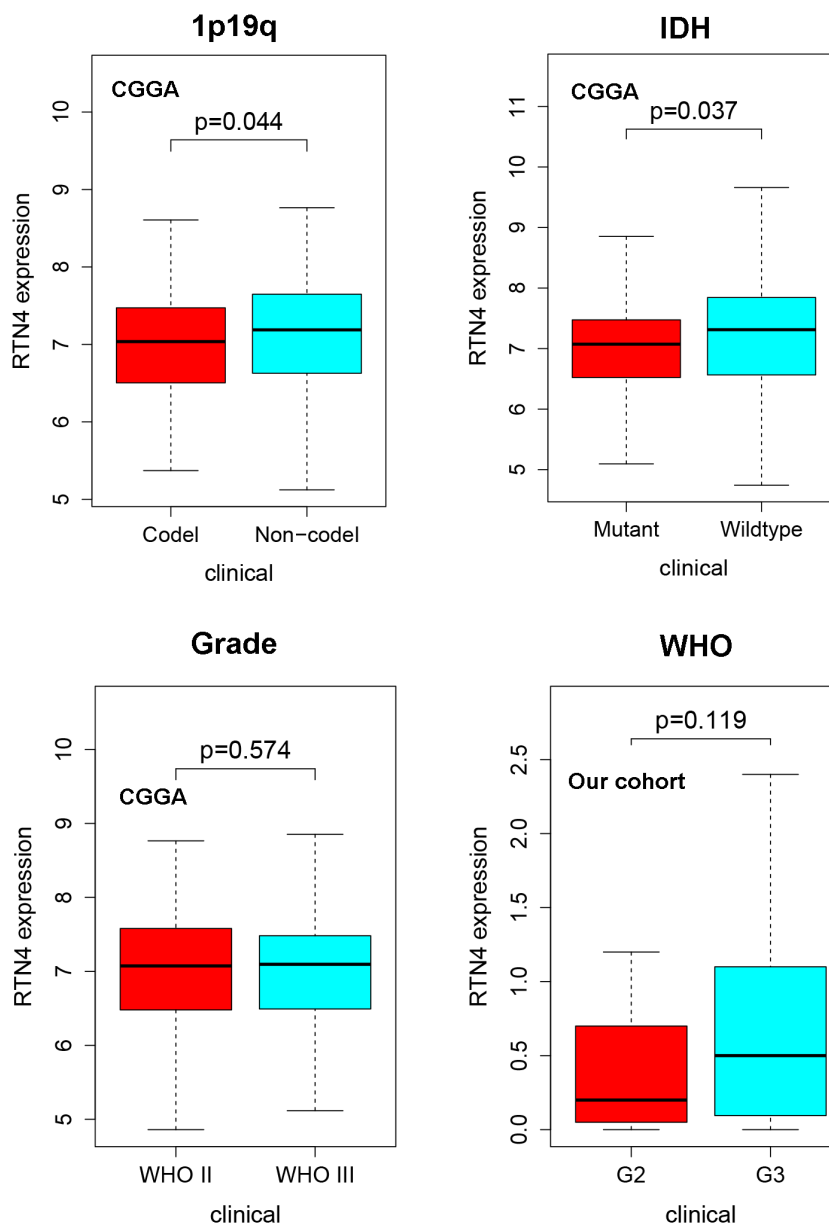

Supplement: Supplementary file 2 — Figure S2. Expression of RTN4 in LGG in CGGA. (A) High RTN4 expression in CGGA is associated with poorer OS. (B) Correlation between RTN4 expression and clinical features. [file JCMM-29-e70418-s005.pdf]

**A**

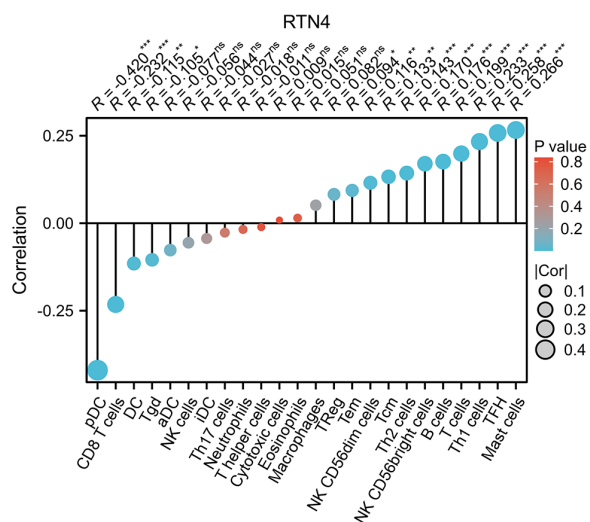

**B**

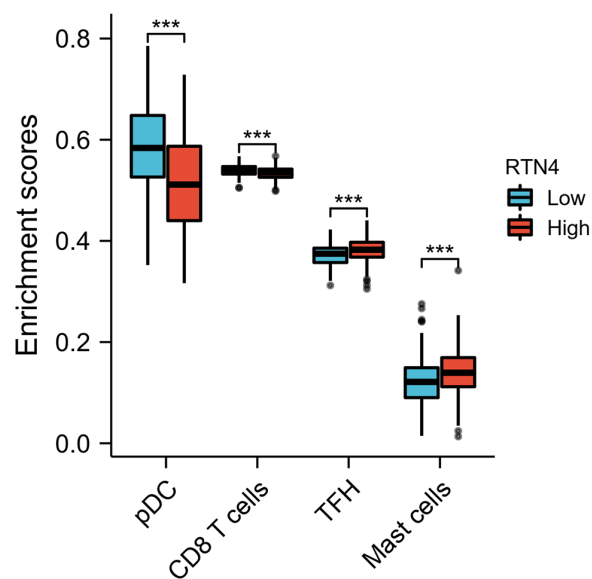

**C**

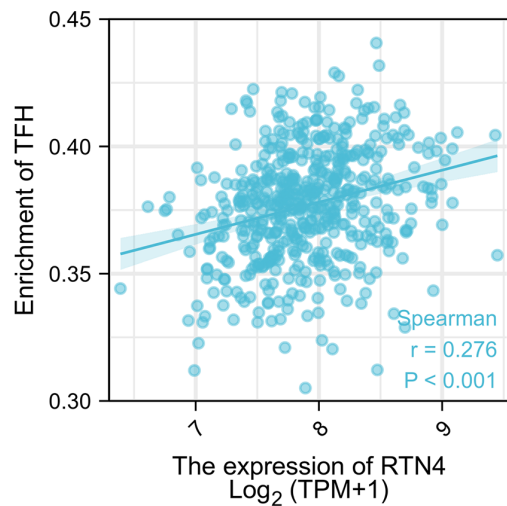

## D

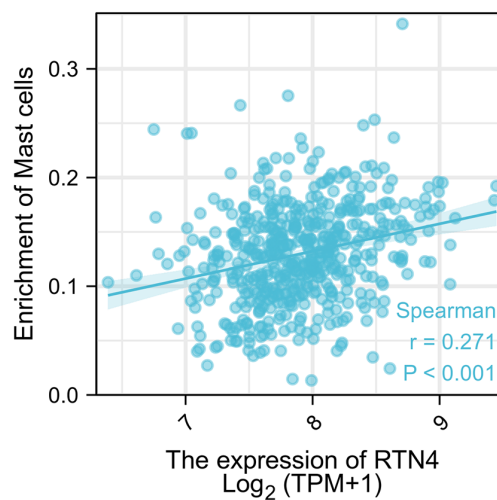

**E**

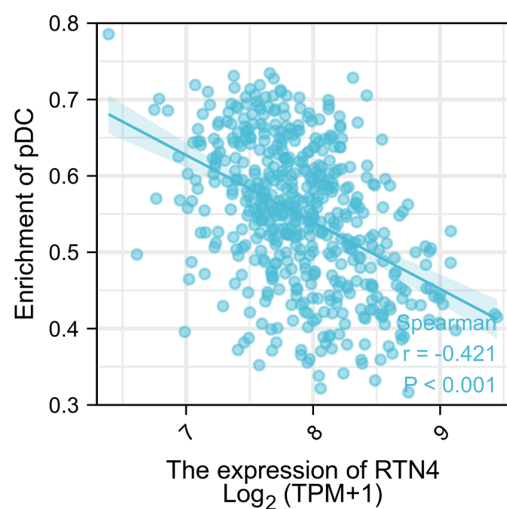

**F**

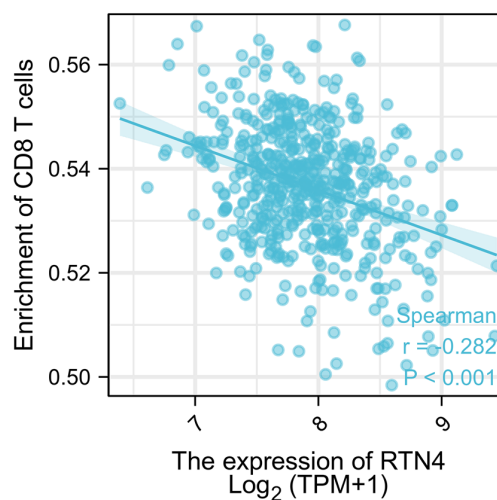

Supplement: Supplementary file 3 — Figure S3. Correlation analysis between RTN4 expression and immune cell infiltration (A) Correlation between RTN4 expression and different immune cell infiltration in LGG (B) RTN4 expression. (C–F) Correlation between RTN4 expression and abundance of tumour‐infiltrating immune cells in LGG. [file JCMM-29-e70418-s001.pdf]
