# Supplementary material for: The Overexpression of RTN4 Significantly Associated With an Unfavourable Prognosis in Patients With Lower‐Grade Gliomas
Source: J Cell Mol Med. 2025 Feb 19;29(4):e70418. doi: 10.1111/jcmm.70418 (PMC11837034; doi:10.1111/jcmm.70418)
Supplement: Supplementary file 4 — Table S1. Patient characteristics in TCGA database. [file JCMM-29-e70418-s002.doc]

| Characteristic | Low expression of RTN4 | High expression of RTN4 | p |
| --- | --- | --- | --- |
| n | 255 | 255 |  |
| WHO grade, n (%) |  |  | 0.157 |
| G2 | 111 (24.5%) | 105 (23.2%) |  |
| G3 | 105 (23.2%) | 132 (29.1%) |  |
| IDH status, n (%) |  |  | 0.015 |
| WT | 36 (7.1%) | 58 (11.4%) |  |
| Mut | 218 (43%) | 195 (38.5%) |  |
| 1p/19q codeletion, n (%) |  |  | 0.011 |
| codel | 70 (13.7%) | 98 (19.2%) |  |
| non-codel | 185 (36.3%) | 157 (30.8%) |  |
| Primary therapy outcome, n (%) |  |  | 0.251 |
| PD | 41 (9.3%) | 60 (13.6%) |  |
| SD | 73 (16.6%) | 70 (15.9%) |  |
| PR | 32 (7.3%) | 30 (6.8%) |  |
| CR | 71 (16.1%) | 63 (14.3%) |  |
| Gender, n (%) |  |  | 0.533 |
| Female | 110 (21.6%) | 118 (23.1%) |  |
| Male | 145 (28.4%) | 137 (26.9%) |  |
| Race, n (%) |  |  | 0.865 |
| Asian | 3 (0.6%) | 5 (1%) |  |
| Black or African American | 10 (2%) | 11 (2.2%) |  |
| White | 233 (46.7%) | 237 (47.5%) |  |
| Age, n (%) |  |  | 0.092 |
| <=40 | 136 (26.7%) | 116 (22.7%) |  |
| >40 | 119 (23.3%) | 139 (27.3%) |  |
| Histological type, n (%) |  |  | 0.007 |
| Astrocytoma | 108 (21.2%) | 84 (16.5%) |  |
| Oligoastrocytoma | 69 (13.5%) | 59 (11.6%) |  |
| Oligodendroglioma | 78 (15.3%) | 112 (22%) |  |
| Laterality, n (%) |  |  | 0.610 |
| Left | 129 (25.5%) | 119 (23.6%) |  |
| Midline | 3 (0.6%) | 3 (0.6%) |  |
| Right | 120 (23.8%) | 131 (25.9%) |  |
| Age, median (IQR) | 39 (31, 50) | 42 (34, 55) | 0.002 |
