# Supplementary material for: The Overexpression of RTN4 Significantly Associated With an Unfavourable Prognosis in Patients With Lower‐Grade Gliomas
Source: J Cell Mol Med. 2025 Feb 19;29(4):e70418. doi: 10.1111/jcmm.70418 (PMC11837034; doi:10.1111/jcmm.70418)
Supplement: Supplementary file 5 — Table S2. Univariate Cox analysis evaluating independently predictive ability of RTN4 for OS in TCGA database. [file JCMM-29-e70418-s004.doc]

| Characteristics | Total(N) | Univariate analysis | |
| --- | --- | --- | --- |
|  |  | Hazard ratio (95% CI) | P value |
| IDH status | 506 |  |  |
| WT | 94 | Reference |  |
| Mut | 412 | 0.155 (0.107-0.225) | **<0.001** |
| 1p/19q codeletion | 509 |  |  |
| codel | 167 | Reference |  |
| non-codel | 342 | 2.562 (1.602-4.098) | **<0.001** |
| Histological type | 509 |  |  |
| Astrocytoma | 192 | Reference |  |
| Oligoastrocytoma | 128 | 0.614 (0.384-0.982) | **0.042** |
| Oligodendroglioma | 189 | 0.558 (0.374-0.834) | **0.004** |
| Age | 509 |  |  |
| <=40 | 252 | Reference |  |
| >40 | 257 | 2.868 (1.962-4.194) | **<0.001** |
| RTN4 | 509 |  |  |
| Low | 255 | Reference |  |
| High | 254 | 1.502 (1.052-2.145) | **0.025** |
