# Supplementary material for: The Overexpression of RTN4 Significantly Associated With an Unfavourable Prognosis in Patients With Lower‐Grade Gliomas
Source: J Cell Mol Med. 2025 Feb 19;29(4):e70418. doi: 10.1111/jcmm.70418 (PMC11837034; doi:10.1111/jcmm.70418)
Supplement: Supplementary file 6 — Table S3. Univariate Cox analysis evaluating independently predictive ability of RTN4 for OS in clinical samples. [file JCMM-29-e70418-s003.doc]

| Characteristics | Univariate analysis | |
| --- | --- | --- |
|  | Hazard ratio (95% CI) | P value |
| Age | 1.041 (1.011-1.070) | **0.005** |
| IDH status | 1.689 (0.800-3.567) | 0.169 |
| 1p/19q codeletion | 1.485 (0.738-2.990) | 0.268 |
| Tumor Size | 0.701 (0.270-1.817) | 0.464 |
| R0 | 0.366 (0.172-0.777) | **0.009** |
| Radio-status | 0.690 (0.346-1.377) | 0.293 |
| Chemo-status | 0.992 (0.492-2.001) | 0.982 |
| RTN4 | 2.589 (1.351-4.960) | **0.004** |
